# Performance of Colombian Silk Fibroin Hydrogels for Hyaline Cartilage Tissue Engineering

**DOI:** 10.3390/jfb13040297

**Published:** 2022-12-14

**Authors:** Augusto Zuluaga-Vélez, Carlos Andrés Toro-Acevedo, Adrián Quintero-Martinez, Jhon Jairo Melchor-Moncada, Francisco Pedraza-Ordoñez, Enrique Aguilar-Fernández, Juan Carlos Sepúlveda-Arias

**Affiliations:** 1 Grupo Infección e Inmunidad, Facultad de Ciencias de la Salud, Universidad Tecnológica de Pereira, Pereira 660003, Colombia; 2 Departamento de Química de Biomacromoléculas, Instituto de Química, Universidad Nacional Autónoma de México, Ciudad Universitaria, Coyoacán, México City 04510, Mexico; 3 Facultad de Ciencias Agropecuarias, Universidad de Caldas, Manizales 170004, Colombia

**Keywords:** silk fibroin, hydrogels, hyaline cartilage, tissue engineering, chondrogenesis

## Abstract

The development and evaluation of scaffolds play a crucial role in the engineering of hyaline cartilage tissue. This work aims to evaluate the performance of silk fibroin hydrogels fabricated from the cocoons of the Colombian hybrid in the in vitro regeneration of hyaline cartilage. The scaffolds were physicochemically characterized, and their performance was evaluated in a cellular model. The results showed that the scaffolds were rich in random coils and β-sheets in their structure and susceptible to various serine proteases with different degradation profiles. Furthermore, they showed a significant increase in *ACAN*, *COL10A1,* and *COL2A1* expression compared to pellet culture alone and allowed GAG deposition. The soluble portion of the scaffold did not affect chondrogenesis. Furthermore, they promoted the increase in *COL1A2*, showing a slight tendency to differentiate towards fibrous cartilage. The results also showed that Colombian silk could be used as a source of biomedical devices, paving the way for sericulture to become a more diverse economic activity in emerging countries.

## 1. Introduction

Hyaline cartilage is a tissue located at the end of bones and serves as an adequate soft surface for joint movement [1]. Cartilage is an avascular and aneural tissue composed primarily of water, extracellular matrix, and chondrocytes. The last cells are responsible for its synthesis, maintenance, and renewal [2].

The main disease associated with hyaline cartilage damage is osteoarthritis. Knee and hip osteoarthritis is one of the most important causes of chronic disability and pain worldwide and is responsible for substantial financial costs in health care [3]. This is because hyaline cartilage is a tissue with low autoregenerative capacity, and factors such as age and lifestyle affect the outcome [4].

Among the strategies used to recover lost or damaged tissue, those based on tissue engineering, which uses mesenchymal stem cells and scaffolds, stand out for their results. However, even with the latest advances, there are currently no effective treatments available to most people. Therefore, searching for cells and scaffolds for cartilage damage treatment is still ongoing [5].

There are different types of scaffolds or biomaterials, such as films, porous structures, hydrogels, sponges, and membranes, among others [6]. These play a vital role in tissue engineering because they proportion a suitable environment for adequate cell proliferation, differentiation, and regeneration. In other words, these biomaterials act as a temporary extracellular matrix and should therefore be biodegradable and safe for adequate tissue regeneration [7].

The structural and physicochemical characteristics of the biomaterial have an impact on the quality of the graft. However, it is still challenging to develop functionally equivalent scaffolds with relevant clinical results [8]. One of the possible causes is a lack of consistency concerning critical aspects, such as the source of the design and the cell [9].

Many scaffolds are fabricated from biological compounds such as proteins, polysaccharides, or polyesters. An example is silk fibroin-based scaffolds, a fibrillar protein extracted from some arthropods, such as *Bombyx mori*, composed of a heavy and a light chain bound by disulfide bridges [10]. Silk fibroin is considered an advantageous choice for tissue engineering because of its superior mechanical properties, highly organized structures, and low degradability, allowing for the correct formation of new tissue [11]. Their broad usability is additionally related to properties such as low toxicity, oxygen and nutrient permeability, and low immunogenicity. Silk fibroin offers exceptional benefits over conventional biomaterials regarding cell adhesion and growth due to a stable microenvironment attributable to the hydrogen bonds formed between repetitive amino acid sequence, its hydrophobic nature and high crystallinity [12].

Silk fibroin-based hydrogels are manufactured by subjecting fibroin solutions to vortexing, sonication, electric current, pH changes, or the addition of cross-linkers [13]. The proper characteristics of hydrogels, such as tolerance by tissues, their capacity for molecular diffusion, and their compatibility with bioactive agents, such as cells and growth factors, have led to increased interest in their application in regenerative medicine.

In the last decade, research on the use of silk fibroin for cartilage treatment has increased significantly [14,15,16]. However, even if it is a highly versatile biopolymer, only a few medical products are used in clinical routines [17]. In Latin America, this is more critical; when searching in Scopus and PubMed for search criteria (HYALINE CARTILAGE and SILK FIBROIN) focusing on Latin America, the resource is used only in one article. However, a commercial silk fibroin solution was employed [18]. In this sense, it becomes fundamental to generate products and research using prime materials from local regions, with the objective of potentializing the industrialization and the generation of value chains from agricultural practices such as sericulture.

In Colombia, commercial silk is obtained only from the Pílamo 2 hybrid silkworm cocoons. This hybrid was developed by classical genetic improvement through crosses of breeds that could adapt to the environmental and climate conditions of Colombia. Recently, our research group explored the mechanical characteristics of Colombian hybrid silk fibroin hydrogels, finding that the Young modulus and the rheological behavior satisfied the necessities of tissues with high mechanical requirements [19].

All this considered, this work aims to evaluate the performance of silk fibroin hydrogels fabricated from the cocoons of the Pílamo 2 Colombian hybrid in the regeneration of hyaline cartilage. To achieve this, the scaffolds were physicochemically characterized, and their performance was evaluated in an in vitro model.

## 2. Materials and Methods

### 2.1. Obtention of Sterile Silk Fibroin Solution

Colombian hybrid silkworm cocoons (*Bombyx mori* L. var. Pílamo 2) were obtained from the experimental farm “El Pílamo”, administered by the Universidad Tecnológica de Pereira, Colombia. The silk fibroin solution was prepared following the protocol described by Kaplan et al. [20]. Briefly, 5 g of silk cocoons were selected and degummed by boiling in 0.02 M Na_2_CO_3_ for 30 min. After three washes with deionized water, silk fibroin was dried at 45 °C for 8 hours in an oven. The silk fibroin was then solubilized at a concentration of 20% in 9.3 M LiBr, at 60 °C for 8 hours. The residual solution was dialyzed against deionized water for 54 h using a 6000–8000 MWCO cellulose membrane, changing the water every 6 hours. Dialysis timing was determined by measuring the conductivity in the permeate. The resulting silk fibroin solution was vapor sterilized at 121 °C for 15 min and centrifuged twice at 9000× *g* for 20 min. TGA analysis was used to determine the effect of sterilization on silk fibroin mass. Finally, the concentration of silk fibroin was measured by comparing the mass of the solution with the mass of silk fibroin after drying at 70 °C for 12 h.

### 2.2. Fabrication of Silk Fibroin Hydrogels

Silk fibroin hydrogels were manufactured by mechanical crosslinking, following the methodology established by Zuluaga-Vélez et al. [19]. Briefly, hydrogels were formed by sonication (Microson XL 2000) of 4% silk fibroin sterile solutions at 20 W for 15 s. All solutions were incubated at 37 °C for 1 hour and hydrogel formation was verified by inverting the tube [21].

### 2.3. Scanning Electron Microscopy (SEM)

Samples for SEM analysis were prepared, as reported by Wang et al. [22]. The hydrogels were frozen at −20 °C for 4 hours and then lyophilized for 24 h. After drying, the hydrogels were fractured and coated with gold using a cathode sputterer (Quorum Q3000TD).

### 2.4. Attenuated Total Reflectance Fourier Transform Infrared (ATR-FTIR)

Hydrogels were analyzed on a Cary 630 spectrometer coupled to an ATR modulus (Agilent Technologies). The secondary structure was quantified following the methods proposed by Goormaghtigh [23] and Belton [24] with some modifications. Spectra were loaded into Fityk 0.9.8 software [25], and amide I bands between 1730–1600 cm^−1^ were selected. Using a Levenberg–Marquardt numerical method, the band was adjusted to four normal curves calculating the percentage of its contributing areas. These areas were assigned to the following secondary structures: helices and turns (1682–1645 cm^−1^), parallel β-sheets (1637–1613 cm^−1^), and antiparallel β-sheets (1637–1613 cm^−1^).

### 2.5. Degradation of Silk Fibroin Hydrogels by Serine Proteases

Proteolysis was performed as described by Brown et al. [26] with certain adjustments. The scaffolds were incubated with 500 μL of the enzymatic solutions at 37 °C for 6, 12, 18, and 24 h. After incubation, supernatants were analyzed by FPLC (Biologic Duoflow F10, Biorad) using a molecular exclusion column (AdvanceBio SEC 130 A, 2.7 μm, Agilent) eluting with 20 mM pH 7.0 sodium phosphate buffer in an isocratic flow of 0.4 mL min^−1^ monitoring the absorbance at 214 nm.

For the assay, we used solutions of the following enzymes: proteinase K (Sigma), protease XIV (Sigma), and serralysin (produced and purified in-house, according to Vélez-Gómez et al. [27]). The assay was normalized at 3000 U mL^−1^ using azocasein digestion.

For each incubation time, the chromatographic profiles were corrected by subtracting the chromatographic protease profile from the supernatant chromatographic profile, which resulted from the proteolysis of silk fibroin hydrogels. Additionally, the areas under the curve were analyzed for 24 h enzymatic digestions.

### 2.6. Culture of hMSCs

Mesenchymal stem cells (hMSCs) were cultured in MSCGM medium (Lonza) and incubated at 37 °C, 5% CO_2_. The medium was changed every 2 days. The cell surface profile was tested using the MSC Phenotyping Kit Human (Miltenyi Biotec, Auburn, CA, USA ) on a Guava EasyCyte flow cytometer (Luminex, TX, USA).

### 2.7. Measurement of Cell Metabolic Activity

Evaluation of cell metabolic activity was performed using the Alamar Blue assay [28] using hMSCs. Cells were seeded on a polystyrene plate (control), on silk fibroin hydrogels (2D hydrogel), and inside the hydrogel (3D hydrogel). In the case of 3D hydrogels, cells were seeded 10 min after sonicating the silk fibroin solution. For all experiments, cells were seeded at a density of 1500 cells per well, in 96-well plates. After 24, 48, and 72 h of exposure to the biomaterials, the medium was removed and replaced by 200 μL of fresh media with 10% Alamar Blue. Finally, after 4 hours of incubation at 37 °C, 150 μL of each sample was added to an opaque polystyrene plate, and fluorescence was measured at 565/590 nm (excitation/emission) in a Cary Eclipse (Varian) fluorescence spectrophotometer.

### 2.8. Chondrogenic Differentiation of hMSCs in Silk Fibroin Hydrogels

5 × 10^5^ hMSCs were cultured in hMSC Human Mesenchymal Stem Cell Chondrogenic Differentiation Medium (Lonza) supplemented with TGF-β3 (Lonza) in 15 mL conical tubes. Three types of samples were evaluated in triplicate: cell pellet, cell pellet on silk fibroin hydrogels (2D), and cells encapsulated inside silk fibroin hydrogels (3D). For cell pellet culture, cells were centrifuged at 400× *g* for 10 min [29]. In the 2D culture, 50 μL of the sonicated silk fibroin solution was rested for 30 min before cells were deposited on top and centrifuged at 400× *g* for 10 min. In the case of 3D culture, cells were seeded and mixed by pipetting after 10 min of sonicating the silk fibroin solution (50 μL). The cells were cultured in a medium that was changed every two days for 28 days under standard conditions. The resulting cultures were stained with Alcian Blue. Furthermore, cells were frozen in liquid nitrogen, and their total RNA was extracted using TRIzol Reagent (Life Technologies, New York, NY, USA) to evaluate the expression levels of *ACAN*, *COL2A1*, *COL10A1*, *SOX9*, *COL1A2*, and the housekeeping control gene *GAPDH*. Total RNA was diluted in water, quantified using a NanoDrop2000, and kept at −80 °C. RT-qPCR was performed with the One-Step RT-PCR Kit and TaqMan Gene Expression Assays (Applied Biosystems, Foster City, CA, USA) using the 2^−ΔΔCt^ method [30], taking hMSCs as a control.

### 2.9. Statistical Analysis

The results are shown as the mean value ± standard deviation (SD), and the experiments were carried out with at least three replicates. Statistical tests were conducted using GraphPad Prism, version 6.0 (GraphPad Software). *p* < 0.05 was considered significant.

### 2.10. Ethics Approval

The study was approved by Comité de Bioética at the Universidad Tecnológica de Pereira (Code: 04-180520, 20 May 2020). Human Mesenchymal Stem Cells (hMSCs) were obtained from Lonza Bioscience, Basel, Switzerland, Catalog: PT-2501).

## 3. Results and Discussion

### 3.1. Obtention of Colombian Silk Fibroin Solution

**Figure 1:**
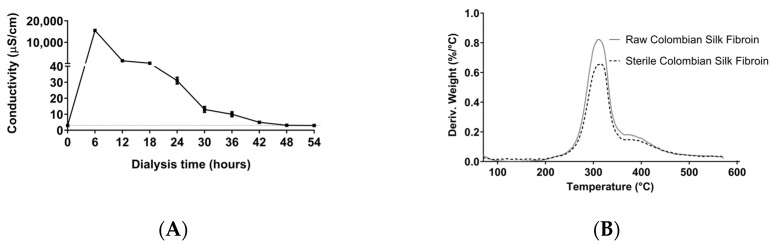
Purification and thermal stability of Colombian silk fibroin. (**A**) Monitoring of silk fibroin solution during dialysis. (**B**) Thermogravimetric analysis of raw and sterile silk fibroin.

The silk fibroin solution was obtained from the Colombian hybrid silkworm “Pílamo 2”. Although there are different silk fibroin fiber dissolution systems, such as organic acid complex compounds, ionic liquids, cuoxam metal complex systems, inorganic acids, alkalis combined with enzymes, and organic compounds, we used lithium bromide for silk fibroin solubilization. Lithium bromide is an expensive reagent, but it is neutral and does not hydrolyze the silk fibroin peptide bonds [31], key for the correct formation of hydrogels. However, the literature does not monitor lithium bromide residues in the protein solution. Often, the analysis of possible contaminants is associated with FTIR monitoring, which does not detect simple anions and cations. As shown in Figure 1A, we demonstrated that 48 h dialysis with changes every 6 hours was enough to lower the electric conductivity of the permeate to that of deionized water. Even if it is an indirect measurement, this simple classical method allows the detection of lithium bromide with reproducible results [32,33].

**Figure A1:**
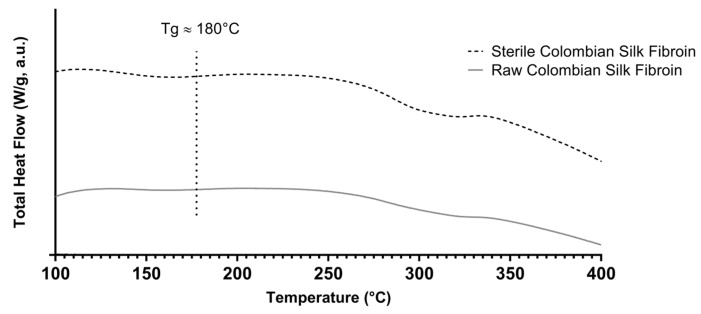
Heat flow curves of raw and sterile Colombian silk fibroin.

Sterile silk fibroin solutions were obtained at a concentration of approximately 7% (*w*/*v*). The thermal analysis demonstrated for the solution that the silk fibroin of the Colombian hybrid is resistant to sterilization temperature (121 °C). The analysis shows that the maximum local degradation rate after sterilization did not change, and it took place at around 311 °C (Figure 1B). These values are consistent with what was previously reported in the literature [34] and allow silk fibroin biomaterials to be safely sterilized using simple methods, such as water vapor. As shown in Figure A1, the total heat flow for raw and sterile silk fibroin displayed a tiny downward signal at ∼180 °C (T_g_), a typical endothermic annealing peak representing the transition of amorphous silk fibroin from hard to soft. These values are close to those reported by Kasoju et al. [35], who established that the flatness of the total heat flow curves could be attributed to the highly ordered crystalline structure of silk fibroin fibers.

### 3.2. Characterization of Colombian Silk Fibroin Hydrogels

**Figure 2:**
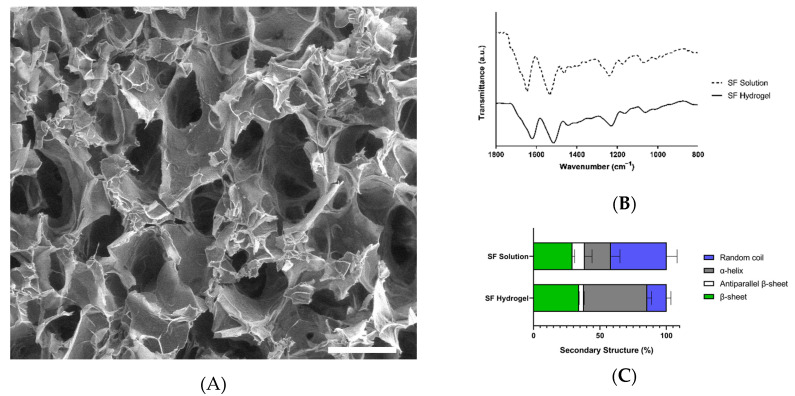
Physicochemical characterization of Colombian silk fibroin hydrogels. (**A**) SEM image of a Colombian silk fibroin hydrogel. The bar measures 200 μm. (**B**) FTIR spectra of Colombian silk fibroin in solution and hydrogel. (**C**) Secondary structure analysis of Colombian silk fibroin solution and hydrogel from deconvolution of FTIR spectra. The error bars indicate standard deviations of the measurements in triplicate. Data were analyzed using the ANOVA test.

Hydrogels were successfully formed through mechanical cross-linking. SEM showed that all hydrogels formed a network of interconnected pores without large aggregates (Figure 2A). In some cases, sonication induced a heterogeneous dispersion of the biomaterial, which may lead to the leaching of silk fibroin and influence the biological response [36].

ATR-FTIR analysis showed that the silk fibroin solution and hydrogels possessed the characteristic amide bands, i.e., amide I (1630–1650 cm^−1^), amide II (1520–1540 cm^−1^) and amide III (1200–1350 cm^−1^), which are associated with the lengthening of the C=O, C-H, and C-N bonds, the bending of the N−H bonds, and to side chain vibrations, respectively (Figure 2B) [37,38]. During gelation, the amide I band changed from 1650 to 1625 cm^−1^, resulting in a change in the way polypeptide chains are packed due to a sol–gel transition, in which hydrophobic interactions between chains form stacking β-sheet structures, while the proportion of less ordered structures decreases, as previously demonstrated [38,39].

A deconvolution of the amide I band was performed to determine the secondary structure of silk fibroin in solution and in hydrogels. Results analysis (Figure 2C) showed that the secondary structure of the silk fibroin in solution behaves similarly to a recently reported regenerated silk fibroin, in which β-sheet and random coil structures are predominant [40]. Silk fibroin from Colombian silkworm *Bombyx mori* L. in hydrogels is dominated by random coils (46–54%), which is consistent with the previously reported low crystallinity of silk fibroin in hydrogels.

β-sheets were the second most abundant secondary structure in hydrogels, induced by sonication and the interactions between domains rich in the small amino acid residues Ala and Gly [41]. It would be expected that β-sheet content would impact mechanical performance, but authors such as Kambe et al. found a nonsignificant positive correlation with the compressive module [42]. Additionally, other authors determined that β-sheet content can be an important factor in the physical properties of hydrogels only when it is uniformly distributed and small. This is achievable when using cross-linking agents, such as horseradish peroxidase (HRP)/H_2_O_2_ [43].

The formation of hydrogels in the present work was achieved through a sol–gel transition by sonication. As previously reported by Kaplan et al. [38,44], this transition is driven by inter- and intramolecular interactions that include hydrogen bonds and hydrophobic interactions occurring mainly through the exposed motif -Gly-Ala-Gly-Ala-Gly-Ser-. Gelation is also enhanced when cysteine residues react with hydrogen and hydroxyl radicals generated when the protein undergoes mechanical scission and forms disulfide bonds.

### 3.3. Biodegradation of Colombian Silk Fibroin Hydrogels

**Figure 3:**
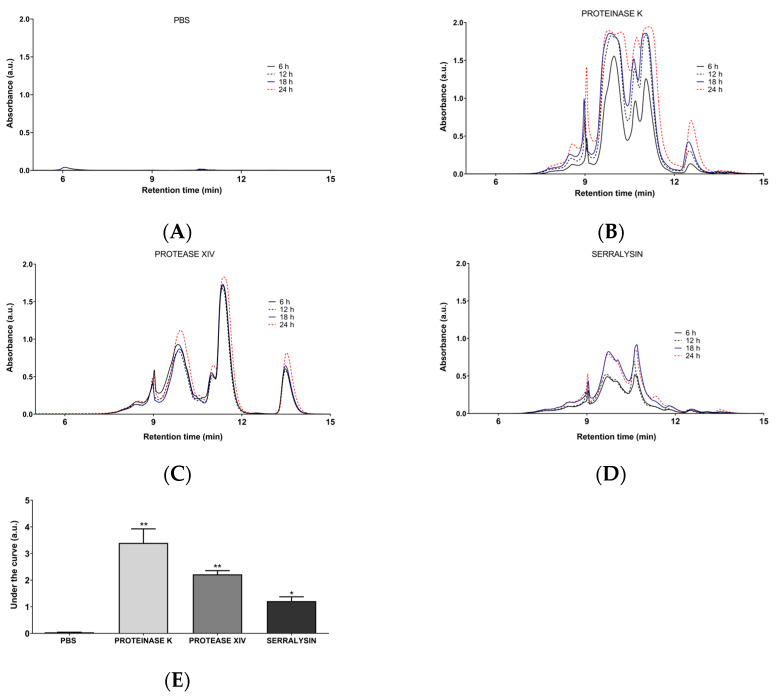
Chromatographic profiles of the supernatant after enzymatic degradation of Colombian silk fibroin hydrogels at 6, 12, 18, and 24 h, monitored at a wavelength of 214 nm: (**A**) Control (PBS); (**B**) Proteinase K; (**C**) Protease XV; (**D**) Serralysin. (**E**) Area under the curve for the enzymatic degradation profiles at 24 h. Error bars indicate standard deviations of triplicate measurements. Kruskal–Wallis test followed by a post hoc Dunn test. (*, *p* < 0.05); (**, *p* < 0.01).

In Figure 3A, the absence of signal associated with the peptide bond (A_214_) in the chromatographic profile of the supernatant leached by the hydrogel without proteases showed that the silk fibroin hydrogels were stable for 24 h. This demonstrates that the mechanical cross-linking process was adequate, confirming the results of the physicochemical characterization. In the presence of proteases (Figure 3B–D), the biodegradation of silk fibroin hydrogels only slightly increased with time. This may be related to the stability of silk fibroin hydrogels and possibly to enzyme autoproteolysis [45].

The three enzymes did not appear to have distinct targets and resulted in similar degradation patterns in terms of molecular weight, possibly related to the highly repetitive sequence of silk fibroin, rich in (Gly-Ala-Gly-Ala-Gly-Ser)_n_ and (Gly-Ala-Gly-Ala-Gly-Tyr)_n_ motifs [41].

Figure 3E shows that Colombian silk fibroin hydrogels are significantly susceptible to proteinase K and protease XIV degradation. This agrees with Brown et al., who established that this susceptibility is caused by the secondary structure of the protein in the biomaterial [26]. In contrast, silk fibroin hydrogels were not highly susceptible to serralysin degradation, opening opportunities for controlled degradation of the biomaterial or the generation of biomaterials with desirable biological properties since serralysin has been shown to possess anti-inflammatory, fibrinolytic, mucolytic, and antimicrobial activities [46].

To the best of our knowledge, this is the first time that size exclusion chromatography has been used to analyze the enzymatic degradation of silk fibroin hydrogels. This technique has been used regularly to study the thermal degradation of silk fibroin [47] and to determine the molecular weights of solutions based on silk fibroin [48]. Unlike SDS-PAGE, size exclusion chromatography allows fractions with different molecular weights to be collected in their native state, which could be used in future biological activity assays with hydrolyzed silk fibroin peptides, similar to what has been constructed with silk sericin [49].

The evaluation of the biodegradability of a biomaterial is a keystone process directly related to its biocompatibility, mechanical stability, and capacity for de novo tissue formation and tissue regeneration. Silk fibroin biomaterials have been observed to degrade slowly, both in vivo and in vitro [50], even in an adjustable fashion [51], allowing correct cell differentiation and the formation of an extracellular matrix before the graft structure collapses.

### 3.4. Cell Metabolic Activity in Colombian Silk Fibroin Hydrogels

**Figure A2:**
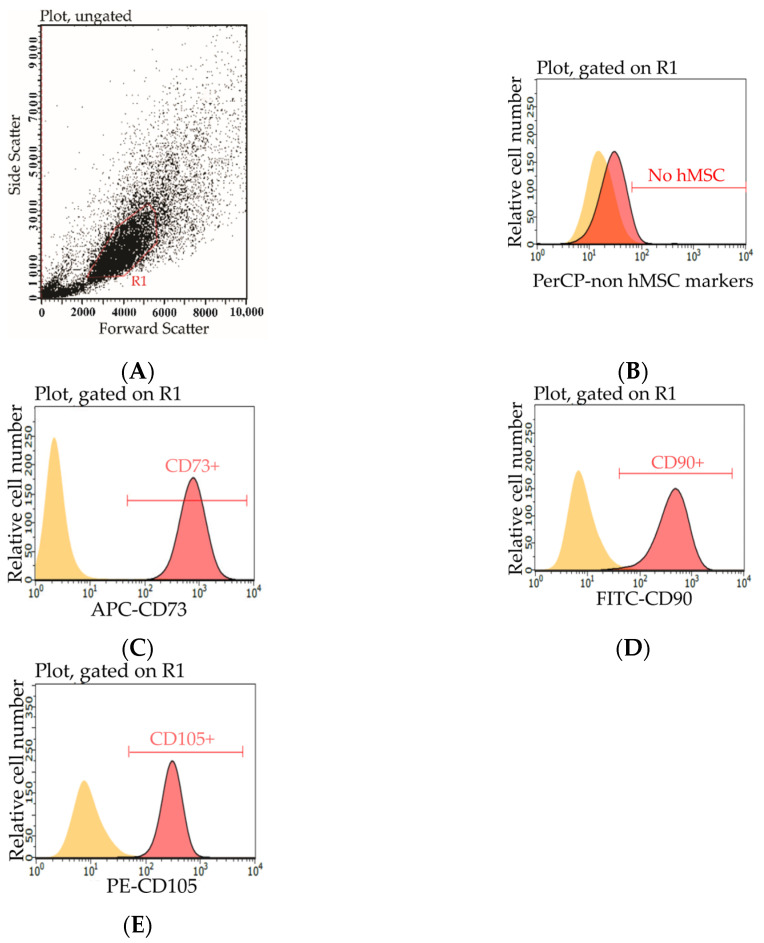
Phenotypification of hMSCs. (**A**) Dot plot of Forward Scatter vs. Side Scatter of ungated data shows hMSCs in region R1. Flow cytometry histogram showing expression of (**B**) CD34, CD45, CD14, CD19, and anti-HLA-DR; (**C**) CD73; (**D**) CD90; (**E**) CD105. Each histogram (red) was overlaid with the corresponding isotype control (yellow) to identify positively stained cells (**B**–**E**).

Mesenchymal cells showed an adequate phenotype according to the minimal criteria established by the International Society for Cellular Therapy. They presented fibroblast-type morphology, were adherent, showed molecular markers CD73, CD90, and CD105 on their surface, and did not possess other transmembranal proteins such as CD45, CD34, CD14 (CD11b), CD19 (o CD79a) or HLA–DR (Figure A2).

**Figure 4:**
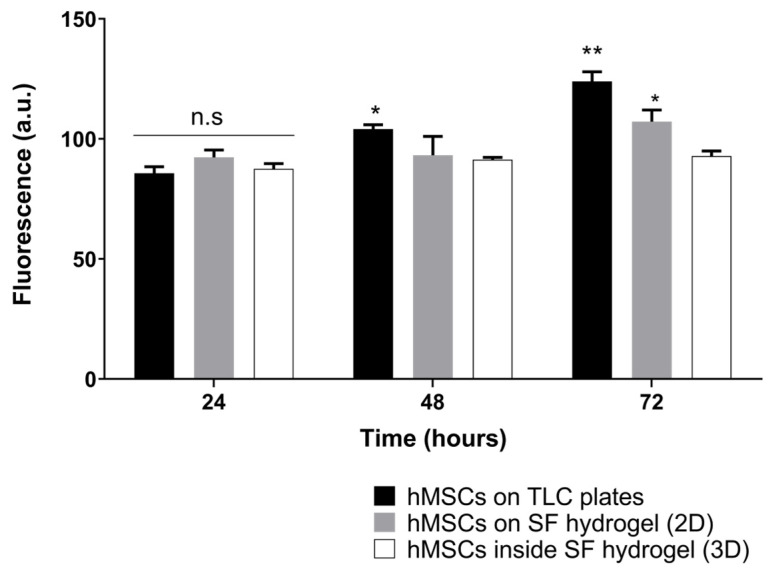
Influence of Colombian silk fibroin hydrogels on hMSC metabolic activity after an incubation period of 24, 48 and 72 h, determined by the Alamar Blue assay. The error bars indicate the standard deviations of quadruplicate measurements. Data were analyzed using two-way ANOVA followed by a post hoc Tukey test. (n.s., nonsignificant); (*, *p* < 0.05); (**, *p* < 0.01).

Figure 4 shows the Alamar Blue results from silk fibroin hydrogels. It was clear that there were no significant differences between the two dispositions of cells in the biomaterials at low exposure periods, which is related to a good process of cell encapsulation. However, after longer expositions, the cell growth rate was higher in the bi-dimensional culture. There was no maim in the fluorescence as time passed, which indicates either a low or nonexistent cytotoxicity in bi- and tridimensional hMSC cultures.

Other researchers have reported successful encapsulation of mesenchymal stem cells in 4% silk fibroin hydrogels 10 min after sonication [52]. They reported a slight decrease in resazurin levels in the first days of culture but observed significant cell proliferation after the fifth day. This could be related to contact-induced growth inhibition, as the high density of the polymeric network makes processes such as cell migration and nutrient diffusion more challenging [53].

Although desired, the proliferation of hMSCs does not guarantee the success of the construct. Cell proliferation and differentiation are inversely related [54], implying that cells continue to divide before fully differentiating to chondrocytes, through transcription factors and regulators such as cyclin-dependent kinase 1 (Cdk1) [55].

### 3.5. Chondrogenesis in Colombian Silk Fibroin Hydrogels

**Figure 5:**
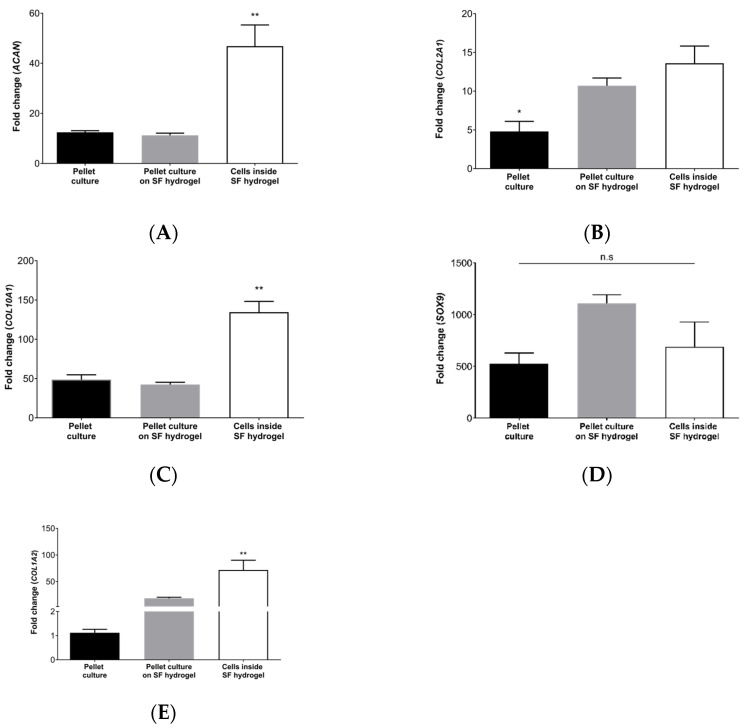
Chondrogenic gene expression profiles: (**A**) *ACAN*; (**B**) *COL2A1*; (**C**) *COL10A1*; (**D**) *SOX9*. (**E**) *COL1A2*. Error bars indicate standard deviations of triplicate measurements. Kruskal–Wallis test followed by a post hoc Dunn test. (n.s., nonsignificant); (*, *p* < 0.05); (**, *p* < 0.01).

Chondrogenic genes were clearly expressed after 28 days of culture, demonstrating successful cell differentiation (Figure 5). Our results also showed that pellet culture is an adequate standard for chondrogenesis, avoiding animal models in the preliminary proof of biomaterials. This type of culture mimics the structure, composition, density of ECM, and cell distribution of native hyaline cartilage [56]. The analysis of gene expression showed that cells adapted to silk fibroin hydrogels, evidenced by a significant increase in *ACAN* and *COL2A1* compared to the pellet culture alone. These two genes are directly related to the microarchitecture of the ECM of hyaline cartilage, which is fundamental for the tissue’s mechanical and biological properties of the tissue. *COL2A1* expression is related to the synthesis of type II collagen fibrils that characterize ECM and limit chondrocyte hypertrophy [57]. *ACAN* expression is implicated in the production of aggrecan, a fundamental proteoglycan in the water catchment, essential for the distribution of mechanical charge on the surface of the joint [58].

*COL10A1* was highly overexpressed in hMSCs differentiated inside silk fibroin hydrogels, which has been previously reported for other types of scaffolds [59]. Although *COL10A1* is generally associated with hypertrophic cartilage, it is also a critical component of endochondral bone formation during skeletal development and the regeneration of osteochondral lesions [60].

*SOX9* was overexpressed in all samples compared to hMSCs cultured in a nonchondrogenic medium. This gene is a master regulator of chondrogenesis, regulating the differentiation of hMSCs into chondrocytes and promoting cell proliferation. Its expression is related to the regeneration of hyaline cartilage [61].

The soluble portion of the silk fibroin hydrogels did not affect chondrogenesis. This suggests that the biomaterial degrades safely without producing chondrogenesis inhibitors. Some authors have described that degradation could even increase the content of type II collagen and improve nutrient diffusion [62].

An unexpected result was that silk fibroin hydrogels promoted differentiation to fibrous cartilage (increased *COL1A2*). This finding could negatively affect hyaline cartilage regeneration because this would result in mechanically inferior fibrocartilage [63]. This suggests that Colombian silk fibroin hydrogels could be helpful in the regeneration of other tissues, such as the intervertebral disc, a tissue rich in proteoglycans and types I, II, III, and X collagens [64]. This tissue has already been treated with biomaterials based on this protein, confirming its versatility [65].

A limitation of our study is the absence of information concerning the abundance of chondrogenic proteins using immunofluorescence or Western blotting techniques. However, several studies support the fact that differentially expressed mRNAs directly correlate with their protein product, although there might be a slight delay [66,67]. In the case of hyaline cartilage, proteomic and transcriptomic analysis confirm a strong enough tissue-specific mRNA-protein correlation to monitor the progression of osteoarthritis [68] or the regeneration process [69].

**Figure 6:**
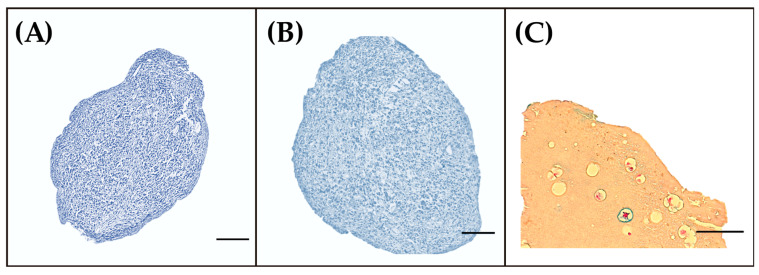
Alcian Blue stain of (**A**) pellet culture; (**B**) pellet culture on Colombian silk fibroin hydrogel; (**C**) cells inside Colombian silk fibroin hydrogel. The bars measure 50 μm.

Alcian Blue staining can be observed in Figure 6. The images show that sulfated glycosaminoglycans were deposited close to the cells, represented by a light blue color. This indicates adequate cell differentiation and is associated with correct water collection that can mechanically stabilize the graft, through the distribution of mechanical loads [70].

Pellet cultures (Figure 6A,B) show an adequate cell distribution, with a region composed of round chondrocyte-like cells, with lacunae and isogenic cell groups, supporting the results of RT-qPCR. Figure 6B shows that neither the soluble moiety of the hydrogel nor the cell–hydrogel interaction affected the production of GAGs. It is important to note that a homogeneous distribution of cells was not achieved in the encapsulation process (Figure 6C). This generates discontinuous distributions of extracellular matrix in the construct, as mentioned in Section 3.2.

Glycosaminoglycan production was highly evident in the pericellular space, with low diffusion of molecules through the material, which could be related to hydrogel porosity [71]. These results agree with those of Vunjak-Novakovic et al. [72], who demonstrated that recently synthesized glycosaminoglycans are located in silk fibroin microfibers.

Chondrogenic differentiation assays agree with the results of other authors that constructed 4% hydrogels from other sources of silk fibroin [73,74,75]. This demonstrates the high potential of silk fibroin hydrogels obtained from the Colombian hybrid Pílamo 2 for human hyaline cartilage regeneration. It creates an opportunity to develop affordable medical devices in emerging countries such as Colombia.

## 4. Conclusions

Silk fibroin hydrogels from the Colombian hybrid can be fabricated under sterile and stable conditions, which is fundamental for standardization approaches for biomedical device production. Furthermore, they were shown to be rich in random coils and β-sheets in their structure and susceptible to various serine proteases with different degradation profiles.

The results demonstrated the high potential of Colombian hybrid Pílamo 2 silk fibroin hydrogels for the engineering of hyaline cartilage tissue. However, it is necessary to evaluate the in vivo formation of fibrocartilage and biomaterial performance when there is articular damage. The results also showed that Colombian silk could be used as a source of biomedical devices, which paves the way for sericulture to become a more diverse economic activity in emerging countries.

## Data Availability

Not applicable.
